# Hydroxycamptothecin-loaded nanoparticles enhance target drug delivery and anticancer effect

**DOI:** 10.1186/1472-6750-8-46

**Published:** 2008-05-04

**Authors:** Anxun Wang, Su Li

**Affiliations:** 1 Department of Oral and Maxillofacial Surgery, First Affiliated Hospital, Sun Yat-sen University, Guangzhou, China; 2 Department of Medicine, Tumor Hospital, Sun Yat-sen University, Guangzhou, China

## Abstract

**Background:**

Hydroxycamptothecin (HCPT) has been shown to have activity against a broad spectrum of cancers. In order to enhance its tissue-specific delivery and anticancer activity, we prepared HCPT-loaded nanoparticles made from poly(ethylene glycol)-poly(γ-benzyl-L-glutamate) (PEG-PBLG), and then studied their release characteristics, pharmacokinetic characteristics, and anticancer effects. PEG-PBLG nanoparticles incorporating HCPT were prepared by a dialysis method. Scanning electron microscopy (SEM) was used to observe the shape and diameter of the nanoparticles. The HCPT release characteristics in vitro were evaluated by ultraviolet spectrophotometry. A high-performance liquid chromatography (HPLC) detection method for determining HCPT in rabbit plasma was established. The pharmacokinetic parameters of HCPT/PEG-PBLG nanoparticles were compared with those of HCPT.

**Results:**

The HCPT-loaded nanoparticles had a core-shell spherical structure, with a core diameter of 200 nm and a shell thickness of 30 nm. Drug-loading capacity and drug encapsulation were 7.5 and 56.8%, respectively. The HCPT release profile was biphasic, with an initial abrupt release, followed by sustained release. The terminal elimination half-lives (t 1/2 β) of HCPT and HCPT-loaded nanoparticles were 4.5 and 10.1 h, respectively. Peak concentrations (Cmax) of HCPT and HCPT-loaded nanoparticles were 2627.8 and 1513.5 μg/L, respectively. The apparent volumes of distribution of the HCPT and HCPT-loaded nanoparticles were 7.3 and 20.0 L, respectively. Compared with a blank control group, Lovo cell xenografts or Tca8113 cell xenografts in HCPT or HCPT-loaded nanoparticle treated groups grew more slowly and the tumor doubling times were increased. The tumor inhibition effect in the HCPT-loaded nanosphere-treated group was significantly higher than that of the HCPT-treated group (p < 0.01). Tumor inhibition in the control group by PEG-PBLG nanoparticles was not observed (p > 0.05).

**Conclusion:**

Compared to the HCPT- and control-treated groups, the HCPT-loaded nanoparticle-treated group showed a more sustained release, a longer circulation time, increased delivery to tissue, and an enhanced anticancer effect. HCPT-loaded nanoparticles appear to change the pharmacokinetic behavior of HCPT *in vivo*.

## Background

In recent years, microspheres, liposomes, and biodegradable polymers have been used in site-specific drug delivery systems [1-3]. Hydrophilic-hydrophobic diblock copolymers exhibit amphiphilic behavior and form micelles with core-shell architecture. The hydrophobic block forms the inner core, which acts as a drug incorporation site, especially for the hydrophobic drugs. The hydrophilic block forms the hydrated outer shell, which plays a role in preventing uptake by the reticuloendothelial system (RES) [2,4]. The predominant characteristics of these copolymers that have been reported include solubilization of hydrophobic drugs, sustained release, selective targeting, and lower interactions with the RES [2,4,5]. Nanoparticles made from poly(γ-benzyl L-glutamate) (PBLG) and poly(ethylene oxide) (PEG) are hydrophilic-hydrophobic diblock copolymers which have these predominant characteristics [6,7]. Thus, this PEG-PBLG copolymeric carrier may serve as an appropriate vehicle for drug delivery [6,7].

The anticancer activity of camptothecin (CPT) and its natural and synthetic analogs has been shown in a broad spectrum of cancers, including leukemias and cancers of the liver, stomach, breast, and colon [8-10]. Among natural CPTs, 10-hydroxycamptothecin (HCPT) has been shown to be more active and less toxic [8,9,11]; however, natural HCPT is in a lactone form and is water-insoluble. One way to improve the solubility of HCPT is to change the lactone form to the carboxylate form by adding NaOH. However, this leads to less activity and more unwanted toxicity [8,9]. At the same time, HCPT has a short half-life in vivo and poor biodistribution [12].

To improve the solubility of CPT analogs, the lactone form of the analogs was incorporated into liposomes or nanoparticles [13-15]. These delivery systems show favorable pharmacokinetics and biodistribution [13-15]. In the present study, we prepared HCPT-loaded PEG-PBLG nanoparticles and investigated the *in vitro *release, pharmacokinetics, and anticancer effect. Our results showed that HCPT-loaded nanoparticles changed the pharmacokinetic behavior of HCPT *in vivo*. The HCPT-loaded nanoparticles had a more sustained release, a longer circulation time, increased delivery to tissue, and an enhanced anticancer effect.

## Results

### Characteristics of HCPT-loaded nanoparticles

**Figure 1:**
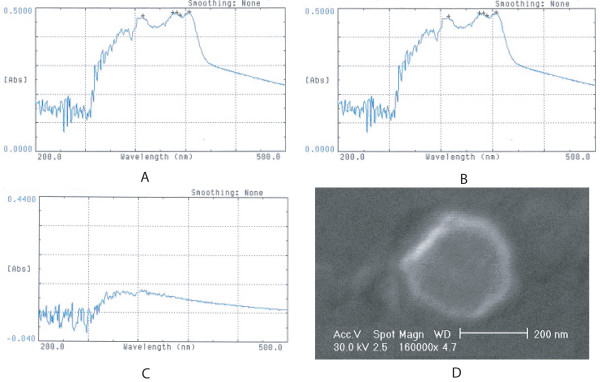
**Characteristics of HCPT-loaded nanoparticles**. (A-C): UV spectrum of HCPT detected by ultraviolet spectrophotometry. At wavelengths of 326 and 368 nm, both the HCPT standard sample (A) and the mixture of HCPT and PBLG/PEG (B) had high absorbency, but the absorbency of HCPT-loaded PBLG/PEG nanoparticles (C) was greatly decreased. (D) The morphology of HCPT-loaded nanoparticles was found under scanning electron microscopy (× 160 000) to be a core-shell structure, spherical or elliptical, with a smooth surface. The hydrophobic central core was a grayish area, approximately 200 nm in diameter. The hydrophilic shell was a bright white ring, approximately 30 nm thick.

In this study, HCPT-loaded PBLG/PEG nanoparticles were prepared using dialysis. After analysis by UV spectrophotometey, we found that the solution of HCPT in a standard sample, or the simple mixture of a solution of HCPT and PBLG/PEG, had high absorbency in the wavelength range, 326 – 368 nm (Fig. 1A,B). However, the absorbency of the solution of HCPT-loaded PBLG/PEG nanoparticles was greatly decreased (Fig. 1C), representing the formation of HCPT-loaded PEG-PBLG nanoparticles. On the basis of absorbency at 326 nm, the drug-loading capacity and drug encapsulation of HCPT-loaded nanoparticles were 7.5 and 56.8%, respectively.

The morphology of HCPT-loaded nanoparticles (Fig. 1D) was found to be a core-shell structure that was spherical or elliptical, with a smooth surface. The hydrophobic central core, the grayish area inside the bright white ring, was approximately 200 nm in diameter. The hydrophilic shell, the bright white ring, was approximately 30 nm in thickness.

### Abrupt-sustained release of HCPT-loaded nanoparticles

**Figure 2:**
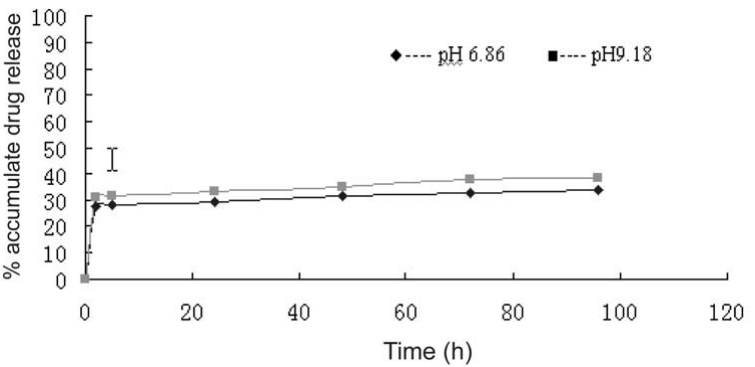
**Release characteristics of HCPT from PEG-PBLG nanoparticles in vitro in different buffers**. The HCPT release profile was biphasic with an initial abrupt release, followed by sustained release. The abrupt release occurred at 2 h and 1/3 of the loaded-HCPT was released during this period. Then, the HCPT entered the sustained release; after 96 h, 3/5 of the loaded-HCPT was still enwrapped in the nanoparticles. In this release study, HCPT-loaded nanoparticles also showed a quick release pattern in the alkaline condition.

The standard curve of a HCPT solution was derived from the following equation: y = 10.7 x + 0.0056 (r = 0.9999). HCPT release from PEG-PBLG nanoparticles at pHs 6.86 and 9.18 in vitro is shown on Fig. 2. The HCPT release profile was biphasic, with an initial abrupt release, followed by sustained release. Abrupt release occurred at 2 h, and 1/3 of the loaded HCPT was released by that time. Then, the release of HCPT entered sustained release following initial abrupt release. After 96 h, 3/5 of the loaded HCPT was still enveloped in the nanoparticles. In this release study, HCPT-loaded nanoparticles showed a quicker release pattern in the more alkaline condition.

### Pharmacokinetic characteristics of HCPT-loaded nanoparticles

**Figure 3:**
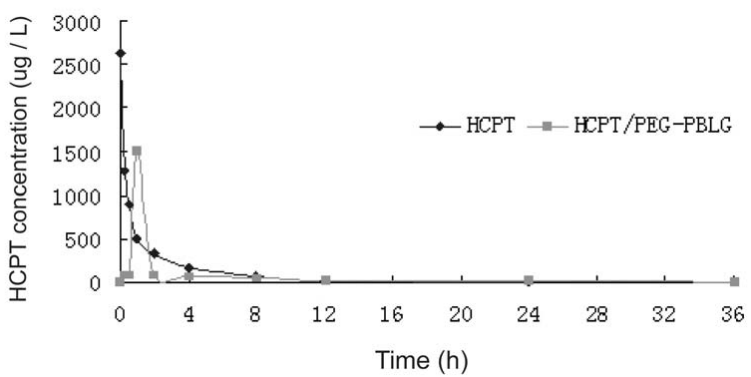
**The mean plasma concentration of HCPT after IV administration of HCPT or HCPT/PEG-PBLG nanospheres at a single dose of 12 mg/kg**. The HCPT release profile from HCPT-loaded nanoparticles showed a biphasic with an initial abrupt release, followed by a sustained release. The abrupt release occurred at 1 h and the peak concentration was 1513.5 μg/L. The release then became sustained, with a plasma concentration between 84.7 and 7.4 μg/L.

The standard curve for HCPT in rabbit plasma was derived from the following equation: y = 2.75 x +2.90 (γ > 0.9999), where Y is the concentration (μg/L) and X is the peak height. The limit of determination was 2 μg/L. The mean plasma concentrations over time for the free HCPT and the HCPT-loaded nanoparticles are illustrated in Fig. 3. The HCPT release profile from HCPT-loaded nanoparticles was biphasic with an initial abrupt release, followed by sustained release. Abrupt release occurred at 1 h and the peak concentration was 1513.5 μg/L. During the sustained release, the plasma concentration was between 7.4 – 84.7 μg/L. As shown in Table 1, the pharmacokinetics of HCPT changed after it was loaded into PEG-PBLG nanoparticles. The terminal elimination half-life was longer, the peak concentration decreased, and the apparent volume of distribution increased.

**Table 1:** The pharmacokinetic parameters of HCPT or HCPT-loaded nanoparticles after IV administration at a single dose of 12 mg/kg in rabbit

	t _1/2 _(h)	C_max _(μg/L)	T_max_(h)	V_d_(L)	AUC(μg · h/L)
HCPT	4.5	2627.8	0	7.3	2459.0
HCPT/PEG-PBLG	10.1	1513.5	1	20	2175.9

*T*_1/2β _: terminal elimination half-life; *C*_max_: peak concentration; *T*_max_: peak time; AUC: area under the curve ;*V*_d _: apparent volume of distribution.

### Tumor inhibition effect of HCPT-loaded nanoparticles *in vivo*

**Figure 4:**
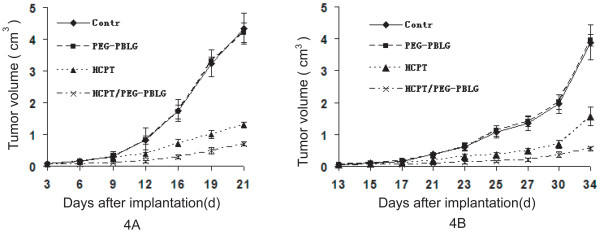
**The tumor growth curve of Lovo cell xenografts (4A) or Tca8113 cell xenografts (4B)**. Xenografts grew quickly in the blank and PEG-PBLG control groups, but growth was significantly slowed in the HCPT- and HCPT/PEG-PBLG-treated groups.

Tumor-associated swellings were visible in all the mice. As demonstrated by the tumor growth curve of Lovo (Fig. 4A) and Tca8113 cell xenografts (Fig. 4B), xenograft growth was fast in the blank and PEG-PBLG control groups, but significantly depressed in the HCPT- or HCPT/PEG-PBLG-treated groups. As shown in Table 2, tumor doubling time lengthened with treatment. The inhibition rate for HCPT alone was between 60 and 70%, but was greater than 80% for the HCPT-loaded nanoparticles. The tumor volumes of the HCPT and HCPT-loaded nanoparticle-treated groups were significantly less than those of the blank and PEG-PBLG control groups (P < 0.01). There was also significantly more tumor inhibition in the HCPT-loaded nanoparticle treated group than in the HCPT treated group (P < 0.01). However, there was no significant difference in tumor inhibition between the two control groups (P > 0.05). No significant toxicity was observed in any of the groups.

**Table 2:** The anticancer effect of HCPT-loaded nanoparticles in the treatment of xenografts

	Lovo cells xenograft			Tca8113 cells xenograft		
	TDT (d)	IR (%)	TV(cm^3^)	TDT (d)	IR (%)	TV(cm^3^)

Blank control	3.0	0	4.336 ± 0.485	3.5	0	3.888 ± 0.547
PEG-PBLG	2.9	0	4.206 ± 0.308*	3.6	0	3.944 ± 0.179*
HCPT	4.3	70.0%	1.299 ± 0.082^#^	4.5	59.8%	1.564 ± 0.286^#^
HCPT/PEG-PBLG	4.9	83.8%	0.701 ± 0.067^#§^	4.9	85.6%	0.559 ± 0.062^#§^

TDT: tumor doubling time; IR: inhibition rate; TV: tumor volume at day 21 for Lovo cells xenograft or day 34 for Tca8113 cells xenograft

*: compared with blank: *P *= 0.451 or 0.765(front:Lovo xenograft; the latter:Tca8113 xenograft); #: compared with blank: *P *= 0.000;

§: Compared with HCPT: *P *= 0.002 or 0.000(front:Lovo xenograft; the latter:Tca8113 xenograft)

## Discussion

Drug-loaded nanoparticles made from natural and synthetic macromolecular materials that are biocompatible and biodegradable have been used for controlling the release of drugs and changing pharmacokinetics and the targets of drug action [1-3]. Nanoparticles have been used to load anticancer drugs to enhance their anticancer effect and decrease their toxicity [14,15], especially for water-insoluble drugs. Nanoparticles made from PEG and PBLG, which are biocompatible and biodegradable macromolecule materials, have been used to load drugs [6,7]. In this study, HCPT-loaded PEG-PBLG nanoparticles were prepared by dialysis, and the drug loading capacity was 7.5%, which was obviously higher than that of HCPT-loaded polybutylcyanoacrylate nanoparticles (1.22%) prepared by the adsorption-enwrapping method [16].

Like many other copolymer nanoparticles [6,7], the shape of HCPT-loaded nanoparticles was mostly spherical or ellipsoid. Close observation of SEM photographs revealed grayish and bright white profiles in the copolymer nanoparticles, which indicated that they are of the core shell type. The grayish center portion, which was 200 nm in diameter, was assigned to the core of the hydrophobic PBLG, and the bright white ring, which was 30 nm in thickness, was assigned to the shell of the hydrophilic PEG. Studies have revealed that nanoparticles are not easily phagocytized by phagocytes when the thickness of the PEG layer is 10 nm for every 100 nm thickness of micelles [17]. Thus, the size of the nanoparticles was suitable to avoid uptake by the RES.

Numerous data have shown that the release of drugs from nanoparticles is biphasic, with abrupt and sustained release components [18]. Abrupt release includes release of the drug adsorbed at the surface of the nanoparticles or diffused from the polymer matrix. Abrupt release enables the drug to quickly reach effective blood concentrations. Sustained release is the release of the drug that was enwrapped inside the nanoparticles and occurs when the nanoparticles biodegrade, or diffuse from the polymer matrix. Sustained release is advantageous to maintain effective blood concentrations of the drug. In this study, the release profile of the HCPT-loaded nanoparticles also consisted of an abrupt release and a sustained release. The HCPT-loaded nanoparticles also showed a quick release pattern in the alkaline condition in vitro. This may be related to the fact that the lactone form of HCPT converts to the carboxylate form in the alkaline condition and becomes water-soluble.

HCPT is an inhibitor of topoisomerase I (Topo I). Other researchers have revealed that only the lactone form of HCPT can form a stable compound with Topo I and DNA, which may be responsible for its anticancer effect [8]. The carboxylate form of HCPT has a low anticancer effect, high toxicity, and poor stability. Previous pharmacokinetic studies with HCPT have indicated that HCPT has a short half-life, a poor affinity for tissue, and a higher combination rate with plasma protein [12,19]. In this study, we found the same results. Therefore, to improve the anticancer effect and decrease the toxicity of HCPT, many researchers have investigated a new preparation of HCPT [13,15,16]. Williams et al. [15] prepared SN-38- (an active compound of irinotecan) loaded phospholipid-PEG nanoparticles by the solvent-evaporation method, which enhanced the lactone ring stability in the presence of human serum albumin and prolonged the existence of the active drug (lactone form) and the half-life *in vivo*. HCPT-loaded nanoparticles prepared by Zhang [16] were targeted to the liver and had a sustained release effect. In our study, the pharmacokinetic parameters of HCPT changed after it was enveloped in the PEG-PBLG nanoparticles. Compared with HCPT, the pharmacokinetic parameters for the HCPT/PEG-PBLG nanoparticles had the following changes: 1) the terminal elimination half-life increased, 2) the peak concentration decreased, and 3) the apparent volume of distribution increased. These results indicate that the HCPT-loaded nanoparticles have the following characteristics: 1) sustained release, 2) prolonged half-life, and 3) increased affinity to tissue. Therefore, they have the properties of an ideal new preparation of HCPT. The studies of Li et al. [7] and Jeong et al. [6] have also shown that 5-fluorouracil- or adriamycin-loaded PEG-PBLG nanoparticles have similar pharmacokinetic characteristics.

HCPT has known clinical efficacy against a variety of solid tumors in humans [8,9]. In this study, free HCPT inhibited the xenograft growths of colon cancer and oral squamous cell carcinoma, and prolonged the tumor doubling times. These findings were also demonstrated in our previous studies [9,20]. Compared with free HCPT, HCPT-loaded nanoparticles had a higher inhibitory effect on colon cancer and oral squamous cell carcinoma. Williams et al. [15] reported a similar result; after treatment with SN-38-loaded phospholipd-PEG, the anticancer effect against HT-29 colon xenografts was higher compared to CPT-11. The higher anticancer effect of HCPT-loaded nanoparticles may be due to one or more of the following reasons. First, the HCPT-loaded nanoparticles have sustained release, a prolonged half-life, and increase the apparent volume of distribution. These characteristics may increase the exposure time of the drug to tumor tissues. Second, the HCPT-loaded nanoparticles stabilize the lactone form of HCPT, which inhibits the activity of Topo I. The longer the period of stabilization, the stronger the anticancer effect. Third, tumor tissue has an abundant blood supply and tumor cells exhibit higher phagocytotic ability. Together, these characteristics would make nanoparticles more likely to enter the tumor cells and would improve the anticancer effect.

## Conclusion

From this research, we found that PEG-PBLG nanoparticles are useful for the solubilization and sustained release of HCPT. HCPT-loaded PEG-PBLG nanoparticles improved the tissue-specific delivery and the anticancer effect of HCPT by changing the pharmacokinetic behavior of HCPT *in vivo*.

## Methods

### Materials

PEG-PBLG block copolymers were prepared by polymerization of γ-benzyl L-glutamate N-carboxyanhydride (γ-BLG NCA) initiated with mono amine-terminated PEG in a methylene dichloride solution by the method described previously [7]. HCPT (lactone form) powder (> 98.5% purity) and HCPT liquid injection were obtained from Huangshi Lishizhen Pharmaceutical Co. (Hubei, China). All other reagents were of analytical grade. Human colon cancer cells (Lovo cell line) or oral squamous carcinoma cells (Tca8113 cell line) were grown in RPMI 1640 (GIBCO, USA) with 10% fetal calf serum (GIBCO), 100 units/ml penicillin G, and 100 μg/ml streptomycin at 37°C in 5% CO2. New Zealand rabbits (2–3 kg) and SPF BALB/c nude mice, 6–8 weeks of age (20–30 g) were purchased from the Animal Center of Sun Yat-sen University. Animal experiments were performed with the permission of the Animal Ethical Commission of Sun Yat-sen University.

### Preparation and identification of HCPT-loaded nanoparticles

HCPT-loaded PEG-PBLG nanoparticles were prepared by dialysis, as described previously [7]. Briefly, PEG-PBLG diblock copolymer and HCPT (1:1 W/W) were dissolved in N,N-dimethylformamide (DMF), then dialyzed using a dialysis bag (molecular cut-off 3500 g/mol; Spectrum Medical Industries, Inc., Houston, TX) against double-distilled water for 24 h. The solution inside the dialysis bag was centrifuged and supernatant (nanoparticles) was filtered through a 0.45 μm filter. A 640 UV spectrophotometer (Beckman) was used to identify the HCPT-loaded PEG-PBLG nanoparticles at the wavelengths, 200–400 nm. The morphology of nanoparticles was observed using a scanning electron microscope (SEM, HITACHI-600; Japan).

### Drug-loading capacity and drug encapsulation

HCPT-loaded PEG-PBLG nanoparticles were added into the dialysis bag, which was placed in DMF. The solution outside the dialysis bag was stirred at 37°C for 3 h and then the drug concentration was measured using a UV spectrophotometer at 326 nm. Absorbency of the solution (A) was used to calculate the drug-loading capacity and drug encapsulation according to the following formulae: drug loading capacity = *M*_HCPT_/*M*_HCPT/PEG-PBLG _and drug encapsulation = *M*_HCPT_/*M *_drug devoted_, where *M*_HCPT _was the drug content of the detected solution (*M*_HCPT _= *D*_HCPT _× *V*, *D*_HCPT _= *A*_sample_/*A*_standard_) × *D*_standard_, *D: *concentration, *V: *volume), *M*_HCPT/PEG-PBLG _was the quantity of the detected solution of HCPT/PEG-PBLG nanoparticles, and *M*_drug devoted _was the initial quantity of HCPT.

### *In vitro *release

HCPT-loaded nanoparticles were added to a dialysis bag and then introduced into a vial with PBS at different pHs (6.86 and 9.18). The medium was stirred at 94 ± 4 revolutions/min at 37°C. At the indicated time intervals (observed until 96 h), the medium was removed and replaced with fresh PBS. The absorbency of samples of these replaced media was detected by an UV spectrophotometer at 326 nm. The released HCPT in these replaced media at different time intervals was calculated from the standard curve, which was set up in the same way. Then, the release curve of HCPT-loaded nanoparticles was described.

### Pharmacokinetic study of HCPT-loaded nanoparticles in rabbit plasma

Six New Zealand rabbits were randomized into two groups. After 12 hours of fasting, a bolus of the sample, equivalent to 12 mg/kg of HCPT or HCPT-loaded nanoparticles, was injected intravenously into each rabbit. Blood samples were withdrawn from the aural vein at the indicated time intervals. After centrifugation, the plasma supernatant was added to acetic acid (pH 3) to produce the lactone form of HCPT. Then, cold methanol-acetonitrile (1 : 1, v/v) was used to precipitate proteins. After centrifuging at 10,000 r/min for 5 min at 4°C, 50 μL of the supernatant were injected into the high-performance liquid chromatograph (HPLC, HP1100; Agilent) to determine the lactone concentration. The analytical column used was Hypersil C18 (5 μm, ID 4.6 mm × 300 mm). The mobile phase was 0.075 mol/L ammonium acetate buffer (pH 6.4)/acetonitrile (78:22 [v : v]). The column was eluted at a flow rate of 1.0 mL/min at room temperature; and the effluent was monitored spectrofluorometrically with an excitation wavelength of 269 nm and an emission wavelength of 550 nm. The concentrations of HCPT were calculated based on the standard curve, which was set up using standard HCPT solution, and the pharmacokinetic parameters of HCPT distribution were estimated using 3p87 programs, or calculated with open two-compartment models.

### Tumor inhibition effect of HCPT-loaded nanoparticles *in vivo*

The xenograft model of colon cancer was established subcutaneously in the right flank of BALB/c nude mice. After xenografts about 5 mm in diameter formed (third day), the mice were randomly assigned to 4 groups (n = 8) as follows: control, PEG-PBLG, HCPT, and HCPT/PEG-PBLG. Then HCPT or HCPT/PEG-PBLG, 3 mg/kg, was intraperitoneally injected daily for 7 times. In the control group, the same volume of PBS or PEG-PBLG was injected intraperitoneally. Mice were sacrificed on day 21. Tumor size was measured by calipers (length and width) every 3 days. The tumor volume (V = 1/2 length × width^2^) was calculated and the tumor growth curve was generated (y = A e ^k*t*^). The tumor doubling time (*T *= ln2/*K*, k: growth rate) and inhibition rate on day 21 were calculated. The inhibition rate was calculated as follows: (1-the volume change of experiment group/the volume change of control group) × 100%. The establishment, grouping, and treatment protocol of the Tca8113 cell xenograft (oral squamous cell carcinoma) was similar to the Lovo cell xenograft, except for the following differences: 1) treatment began on day 8, 2) the drug was injected every 2 days for 8 times (16 days), and 3) the mice were sacrificed on day 34. The anti-tumor activity was evaluated as described above.

### Statistical analysis

In all cases, experiments were replicated in triplicate and data represent mean ± s.d. (standard deviation). Statistical analysis of the inhibitory effect on tumor growth was performed using one-way analysis of variance. *P *< 0.05 denoted significance in all cases.

## List of abbreviations used

HCPT: hydroxycamptothecin; PBLG: poly(γ-benzyl ι-glutamate); PEG: poly(ethylene glycol); SEM: scanning electron microscope; HPLC: high-performance liquid chromatography; RES: reticuloendothelial system; DMF: N,N-dimethylformamide; t_1/2_: elimination half-life; C_max_: peak concentration; T_max_: peak time; AUC: area under the concentration-time curve; V_d_: distribution volume; TDT: tumor doubling time; IR: inhibition rate; TV: tumor volume at day 21 for Lovo cell xenografts or at day 34 for Tca 8113 cell xenografts.

## Competing interests

The authors declare that they have no competing interests.

## Authors' contributions

AW and SL were responsible for experimental design and completion of all laboratory work presented in this article. The manuscript was drafted by AW. All authors approved and read the final manuscript.
